# Factors Impacting the Use of an Allelochemical Lure in Pome Fruit for *Cydia pomonella* (L.) Monitoring

**DOI:** 10.3390/insects16020172

**Published:** 2025-02-06

**Authors:** Alan Lee Knight, Michele Preti, Esteban Basoalto

**Affiliations:** 1 Instar Biologicals, Yakima, WA 98908, USA; uncfencer76@hotmail.com; 2 Independent Integrated Pest Management consultant and researcher, 48018 Faenza, Italy; 3 Facultad de Ciencias Agrarias, Instituto de Producción y Sanidad Vegetal, Universidad Austral de Chile, Valdivia 5110566, Chile; esteban.basoalto@uach.cl

**Keywords:** codling moth, Tortricidae, apple, pear, kairomone, weed management, sterile moths, organic production

## Abstract

A four-component blend comprising pear ester, DMNT, linalool oxide, and acetic acid (CM4K) was identified as a potent allelochemical lure for both sexes of codling moth (CM), *Cydia pomonella* (L.). Studies conducted from 2020 to 2022 in Washington State (USA) examined factors which could impact the lure’s relative performance. The CM4K lure was effective across a range of mating disruption programs and was equally attractive in monitoring wild and sterile CM. The lure remained attractive for at least 10 weeks. Total catch in traps baited with the CM4K was significantly less impacted than a sex pheromone lure located near mating disruption dispensers and female catches were largely unaffected. Traps with the CM4K lure caught significantly more females and fewer males when placed near clusters of fruits in a trellised orchard. Two factors were found to significantly impact the relative performance of the CM4K to sex pheromone lures: the CM4K lure was only equivalent to sex pheromone lures in pear MD orchards, and apple and pear orchards with vigorous weed growth. This is the first report of a monitoring lure for a tortricid moth being negatively impacted by the background odor of non-host weed species present within an orchard.

## Simple Summary

Studies were carried out with a new lure (CM4K) for codling moth, *Cydia pomonella* (L.), a world-wide pest of apples and pears. This lure does not include a synthetic sex pheromone of the moth (naturally released by females to attract males) as found in other lures, but uses three plant volatiles plus acetic acid and catches both moth sexes. The CM4K lure is highly effective in pome fruit orchards treated with several types of mating disruption dispensers. In a majority of our field trials, the CM4K outperformed the best sex pheromone lure. More importantly, the CM4K lure could provide farm managers with useful information on the mating status of the female moths. This allows them to assess the effectiveness of their mating disruption dispensers, and to decide if they need more and/or to spray more. Two conditions were found that limit the efficacy of the CM4K lure somewhat: it is less effective in pear than apple, and it is less effective in orchards that have vigorous weed growth. In both cases, it still performs as well as a sex pheromone dispenser and female catch is not negatively impacted.

## 1. Introduction

Monitoring codling moth (CM), *Cydia pomonella* (L.), in orchards treated with sex pheromones for mating disruption (MD) is an important component of integrated pest management programs to effectively select and time supplemental insecticide sprays [1]. Unfortunately, the use of MD can have a variable and often significant effect on the use of sex pheromone-based lures to monitor the population dynamics of male tortricid moths in orchards [2]. Allelochemical lures that could perform more, independent of MD, and allow monitoring of both male and female CMs have been suggested as a useful alternative to sex pheromone lures [3]. Improvements in establishing action thresholds and timing larvicidal sprays with female catches have been reported [4,5,6].

Olfactory masking of traps has been suggested as a key factor influencing the effectiveness of pear ester (ethyl (*E*,*Z*)-2,4-decadienoate) as a lure in different CM hosts [3,7]. The initial discovery of pear ester’s activity for the CM was in a walnut orchard characterized by mono- and sesquiterpenes, and not aliphatic esters, which are typical of pome fruits [3]. Pear ester lures in pome fruit were found to be less effective in late-season pear orchards, when pear ester is naturally released by ripening fruit [8]. More effective lures were developed or used in MD orchards by increasing the loading of pear ester [9] or using it in combination with sex pheromone ((*E*,*E*)-8,10-Dodecadien-1-ol), nonatriene ((*E*)-4,8-dimethyl-1,3,7-nonatriene), or acetic acid [10,11,12].

More recently, a four-component allelochemical lure was formulated by adding pyranoid linalool oxide to the blend of nonatriene, pear ester, and acetic acid. This novel combination further increased CM catches 3-fold [13]. The new lure was more attractive than the three-component blend of pear ester, acetic acid and N-butyl sulfide [13,14]. The four-component lure was also the most effective lure in monitoring CM females. Female moth removal with trap grids of 60–120 ha^−1^, used in combination with MD, have reduced levels of CM fruit injury up to 60% [15,16,17]. The four-component lure has been commercialized as PHEROCON^®^ MEGALURE CM DUAL 4K (CM4K) (Trécé Inc., Adair, OK, USA). Field trials showed that linalool oxide in the blend can be substituted out with β-Myrcene or (*E*,*E*)-α-Farnesene without any loss in female or total CM catch, but these blends have not been commercialized [18]. The addition of a UV-A light to delta traps baited with the CM4K lure can further increase moth catches 2–3-fold [19].

Another role that improved adult monitoring of CMs could play is in the assessment of sterile insect release (SIR) management programs [20]. The SIR program in British Columbia, Canada, was established in 1992 to initially eradicate and later to suppress wild populations of the CM throughout this region [21]. Traps play a role in detecting the remaining pockets of wild moths and calculating whether the overflooding ratio of sterile versus wild moths (S:W ratio) can be expected to be effective [22]. Initially, 1 mg sex pheromone lures were used to monitor male moths, and then 10 mg lures, when orchards secondarily incorporated MD. More recently, the use of sex pheromone and pear ester combination lures have been adopted for CM monitoring [23]. However, the use of pear ester plus acetic acid lures has been suggested as a better alternative because of several advantages; they remove fewer sterile males, allow for the detection of wild female moths, and their shorter range of activity improves the measure of S:W ratios [23]. Utilizing lures such as CM4K that can catch additional female moths, both wild and sterile, has not been reported.

Trap to trap variability in the monitoring of the CM is often quite large [24]. Except for height in the canopy, trap placement tends to favor convenience, such as by having nearby borders [25]. However, two studies found impacts on trap performance with sex pheromone and pear ester lures [26,27]. The first study found that growers frequently placed traps within 0.3 m of a MD dispenser applied at 1000 ha^−1^, as it is recommended that both be placed in the upper third of the canopy [26]. Moth catches with pear ester lures were negatively impacted by the trap’s proximity to injured fruits [28]. Similar trap placement effects using the CM4K lure have not been considered.

Initial studies with the CM4K lure in MD-treated orchards in Washington State (USA) found that it outperformed the previously most effective CM lure, which was a combination of sex pheromone, pear ester, and acetic acid [29]. Yet, in two subsequent studies conducted in Italy and Massachusetts (USA), the CM4K lure performed similarly to, and not better than, sex pheromone lures in orchards with or without MD and in apple or pear crops [30,31]. Interestingly, these comparative results are similar to earlier studies assessing pear ester as a lure for the CM in Italy and eastern North America [7,32]. It remains unclear whether there is some difference in olfaction among populations of CM or whether differences in orchard ecosystems and their management could be influential.

Background volatiles both outside and inside a host crop play significant roles in the effectiveness of traps baited with allelochemical lures [33]. In general, the literature presumes that competition of the lure with host volatiles is a key determinant of trap performance within the crop [34]. In many cases, the host plant’s volatiles can mask a specific allelochemical signal comprising a single or blend of volatiles [35,36]. Volatiles released by weeds (non-hosts) have not been considered as factors impacting lure performance within orchards (hosts) [37]. Yet, unlike in many agricultural crops, the biomass and species diversity of weed communities in orchards can be substantial both within and between rows, especially under organic management [38,39]. Weed management practices in the various orchards utilized in previous studies with CM4K were not reported. Often, these studies were conducted in poorly managed conventional or organic orchards because they had high CM population densities [30]. Organic weed management is difficult, especially with under-tree sprinklers, as growers have fewer herbicides compared to conventional farming, and they usually mow the grass cover or use mulching or various tilling methods to manage weeds under the tree row [40]. Avoiding the removal of weeds or the planting of flowering weeds has also been promoted to increase ecosystem services for growers [41,42].

Studies were conducted from 2020 to 2022 in Washington State (USA) to evaluate several factors that could impact the performance of CM4K lure for CM male and female monitoring, including the MD type, the crop, wild versus sterile moths, lure longevity, and the presence of vigorous weed growth in the orchards.

## 2. Materials and Methods

### 2.1. Lures and MD Treatments

Three proprietary lures were used for this study (Trécé Inc., Adair, OK, USA): a red rubber septum (PHEROCON^®^ CM 1X, ‘PH1X’) loaded with codlemone, (*E*,*E*)-8,10-dodecadien-1-ol (the major component of CM female sex pheromone); a PVC matrix (PHEROCON^®^ CM DA COMBO-P, ‘Combo’) loaded with codlemone plus pear ester, (*E*,*Z*)-2,4-ethyl decadienoate, combined with a closed membrane acetic acid co-lure (PHEROCON^®^ AA, ‘AA’); and a second PVC lure loaded with pear ester, (*E*)-4,8-dimethyl-1,3,7-nonatriene, and pyranoid linalool oxide (6-ethenyl-2,2,6-trimethyloxan-3-ol) used in combination with the membrane AA co-lure (PHEROCON^®^ MEGALURE CM DUAL 4K, ‘CM4K’).

Orchards were either left untreated or treated with MD dispensers for CM management. Eight types of MD dispensers were included in the various studies and used at variable rates according to manufacturers’ recommendations [43]. Semios CM Aerosol was loaded with 55.4 g of codlemone per canister (SemiosBIO Technologies Inc., Vancouver, BC, Canada) and Isomate CM Mist was loaded with 23.6 g codlemone per canister (Pacific Biocontrol Corp., Vancouver, WA, USA). All canister dispensers were applied at 2.5 units ha^−1^. Three sex pheromone hand-applied dispensers were included, NoMate CM loaded with 135 mg of codlemone (Scentry Biologicals Inc., Billings, MT, USA), Isomate CM Flex loaded with 95.5 mg of codlemone and 62 mg of 1-Dodecanol and 1-Tetradecanol (Pacific Biocontrol Corp.), and CIDETRAK CM PP (PP = puzzle pieces) loaded with 120 mg codlemone per dispenser (Trécé Inc.). All three dispenser types were applied, with 1000 dispensers ha^−1^. In addition, a microencapsulated sprayable product, CIDETRAK CM MEC loaded with 7% codlemone was applied at 250 mL ha^−1^ (Trécé Inc.). Two commercial dispensers loaded with both codlemone and pear ester (PH/PE) were also included, CIDETRAK CMDA COMBO MESO-A loaded with 850 mg codlemone and 500 mg pear ester and applied at 80 dispensers ha^−1^, and CIDETRAK CMDA COMBO PP with 85 and 50 mg of codlemone and pear ester applied at 500–1000 dispensers ha^−1^, respectively (Trécé Inc.). A third experimental dispenser, the CPD (Concentrated Passive Dispenser), was constructed as a cluster of eight CMDA COMBO MESO dispensers clipped onto a 15 cm circular plastic hanger and was applied at 10 units ha^−1^ (Trécé Inc.).

### 2.2. Field Trials

Trials were performed during 2020–2022 in commercial orchards in three areas of central Washington State, USA: Tieton (46.70° N, −120.76° W), Wapato, (46.24° N, −120.28° W), and Sunnyside (46.32° N, −120.01° W). All trials had a similar experimental design and included 6 to 14 replicates per lure treatment. Larger orchards were subdivided into 2.0–3.0 ha plots, and a maximum of two replicates were established per orchard. Orange delta traps (PHEROCON^®^ VI, Trécé Inc.) were attached to poles and placed in the canopy at ca. 3.0 m of height. Traps were spaced ≥25 m apart and >20 m from the borders of the plots down a single row in the middle of the plot. The three lure treatments were randomized in each plot. Lures were placed in the center of the sticky liner (PHEROCON^®^ VI CLEAN-BRAKE^TM^, Trécé Inc.). The sex pheromone septa lures were replaced every 2 weeks, and the two PVC-plus-AA membrane binary lures were replaced after 8 weeks. Liners were replaced at each trap check unless no moths were caught. Moths on liners were counted and sexed in the laboratory using a stereomicroscope. Trials aiming to test different factors affecting the performance of CM4K lure are detailed below and summarized in Table 1.

#### 2.2.1. Seasonal CM Monitoring in Orchards Under Different Types of MD

Season-long monitoring studies were conducted in pome fruit orchards to compare lure performance across a range of MD tactics from 18 May to 13 September 2021 and 23 May–4 September 2022. This included 35 organic apple orchards comprising nine cultivars and five organic pear orchards planted primarily with ‘Bartlet and ‘D’Anjou’. In addition, 20 apple and 4 pear orchards were farmed conventionally. All MEC sprays, aerosol and solid dispensers were applied by the growers, except for the experimental CPD dispensers. MEC sprays were applied on four dates beginning in late April and then approximately every four weeks into mid-August. Aerosols were placed in a 35 m × 35 m grid starting 25 m from the edge of the orchards. Placement of hand-applied dispensers typically began <5 m from the edge of the block, and they were applied on every row at a spacing of 2 to 3 m depending on their density and row spacing. The low-density hand-applied dispensers MESO and CPD products were deployed with a wider distance between dispensers, always considering each orchard’s size and shape. All orchards were sprayed by the grower with standard management and nutritional programs. Sprays applied for CM varied from 1 to 5 applications of diamide, spinosyn, and neonicotinyl chemistries in conventionally managed orchards. Organic blocks were treated with multiple applications of granulosis virus, horticultural oil, and 0–4 sprays of a spinosyn insecticide.

A new coefficient was devised to estimate the disruption of CM male catching under different types of MD programs. This coefficient was calculated as the ratio of CM males caught in traps baited with the PH1X lure (strongly disrupted) divided by the CM males caught in traps with the non-pheromone CM4K lure. Our hypothesis is that male catches with the CM4K lure would be minimally impacted by the sex pheromone-based mating disruption technologies. Because low catches with the PH1X lure under MD can create excessive variability in the coefficient estimate, the replicates where the CM catch was <2 moths per season were excluded from the analyses. Additional data gathered during the 2020 season was included to add more data for untreated orchards and from orchards treated with either MESO or PP codlemone-based dispensers. These data were collected using the same 2021–2022 trapping protocol from 2.0 ha plots across 5 apple and 2 pear orchards in 2020. Orchards were monitored from 7 April to 25 August. The PP and MESO dispensers were deployed at 800 and 80 dispensers ha^−1^ in three and six replicates in mid-April, respectively. Data for trap performance in the absence of MD were collected from three replicates. Trap and lure maintenance was identical to the two later years, and traps in 2020 were checked on 14 dates.

#### 2.2.2. Monitoring Wild and Sterile CM, 2021

Four organic apple blocks that were contracted to receive weekly releases of sterile CM were monitored with the lure sets from 6 May to 13 August. Sterilized laboratory reared and unsexed *C. pomonella* adults were obtained from the Okanagan–Kootenay Sterile Insect Release Program’s mass-rearing facility in Osoyoos (BC, Canada). Moths were sterilized in a Cobalt-60 irradiator before shipment to Washington state. Sterilized moths were chilled, packaged in cardboard cups, and marked with an internal red dye, which allowed them to be easily differentiated from wild moths. Moths were released via an unmanned drone at a rate of 2000 unsexed moths ha^−1^. All moths caught on liners were separated as sterile or wild, and sterile/wild ratios of each sex were calculated with each lure.

#### 2.2.3. Longevity of the CM4K Lure, 2022

New CM4K lures (only the PVC portion) were transferred from a freezer and placed in groups of 4 per trap in delta traps without sticky liners, then positioned in the canopy of a ‘Delicious’ apple orchard near Wapato on 16 May. Eight unused dispensers were kept in the freezer at −20 °C. Sets of eight lures were removed weekly from the orchard for 12 consecutive weeks (taking one lure per trap) and frozen at −20 °C. A field trial was subsequently established with 104 traps (8 replicates × 13 dates) in an organic apple orchard near Tieton to compare moth catches in traps with lures of different ages (0–12 weeks-old). New traps were baited with the CM4K PVC lure of different ages and a new AA co-lure, and randomized in a Tieton apple block at a spacing of 25 m × 25 m. Traps were deployed from 6 August to 27 August, and the total number of moths was counted.

#### 2.2.4. Impact of Dispenser Proximity to Traps Catch, 2022

Two experiments were conducted to assess the relative impact of the trap-dispenser proximity on moth catches with PH1X and CM4K lures. Traps (N = 7) baited with the PH1X or CM4K lures were placed either <1 m or 2.0–2.5 m from the CIDETRAK CM PP dispensers applied at 1000 ha^−1^ in the first trial. The expected average distance between dispensers applied at this density and traps was 2.0–2.5 m depending on row spacing. This study was established on 25 May and terminated after two weeks on 8 June. Weekly releases of sterile moths were conducted in nearby plots in the orchard.

Traps baited with CM4K lures or PH1X lures in the second trial were placed as replicate pairs on either side of a Isomate CM Mist aerosol dispenser at a 10 m distance. This distance was arbitrarily selected as a reasonable position where a farm manager might not notice the aerosol unit when hanging the trap. An assumption was made that growers would want to avoid placing their traps closer to the aerosol unit. This same experimental design was used with the same two lures, but with traps placed 35 m from the aerosol devices. This distance is the expected maximum distance that traps would be placed from aerosols if they are used at the recommended 2.5 units ha^−1^. In both experiments, 800 sterile CM were released along a transect beginning and ending 10 m before and after the trap location within each replicate. The study was established on 25 May and terminated after two weeks on 8 June.

#### 2.2.5. Impact of Fruit Proximity to Trap Catches, 2022

Two experiments were conducted to examine whether moth catch in traps baited with the CM4K lure was impacted by the proximity to fruit. The first study was conducted in a 5-year-old ‘Envy’ apple block planted at 0.5 × 3.5 m (tree x row spacing), 5-wire trellised with the top wire at 3 m. The orchard was conventional and treated with the CIDETRAK CM PP dispenser at 1000 ha^−1^. Weed management comprised chemical weeding on the row and mechanical mowing between rows, with a weed growth <10–15 cm. Twenty-one traps were placed on 14 August on the trellis at a height of 2.5 m in each of three categories based on the presence of 0, 1–4, or >5 fruits being within 1 m of the trap (N = 63 traps). Due to the age of the block, there were open spaces between trees. Traps which were not placed near fruits were typically placed between non-bearing shoots. Traps were checked weekly for five weeks from 7 August until 11 September.

The second study was conducted in a 30-year-old mixed ‘Golden Delicious’ and ‘Red Delicious’ apple orchard planted at 4.3 × 4.3 m (tree × row spacing), trained with a central leader system. The orchard was organic and treated with 800 CIDETRAK CM PP dispensers ha^−1^. No specific weed management was performed by the grower, and weed growth was >30–40 cm. The grower did not thin the fruit, and the crop load during the study was very heavy. Ten traps were placed using poles at a height of 2.5 m in each of two locations in the canopy with respect to their proximity to fruit clusters, either adjacent (<0.5 m) or 2.0–2.5 m away. Traps were placed on 24 August and retrieved after four weeks, on 21 September.

### 2.3. Statistical Analysis

Statistical analyses of the moth catches were performed with R software v. 4.0.3 [44]. Moth catch data (for both sexes) were analyzed according to the data distribution. Data normality was tested with Shapiro–Wilk’s test (*p* > 0.05); a linear model (LM) was used with normal data, while data found to be close to a normal distribution were transformed using the square root function (sqrt(x + 0.5)). Data which were not normally distributed were found to fit a Poisson distribution and a generalized linear model (GLM) was used. Akaike’s information criteria (AIC) and residuals were both used to select the fitted models in each analysis. A multiple comparison post hoc test was performed on the fitted models (GLHT function from multcomp package) and Tukey’s HSD test (*p* < 0.05) was used to discriminate significant differences among treatments. Treatments with a mean of zero catches were not included in analyses. A coefficient was calculated to compare CM male catch in traps with the PH1X versus CM4K lures for each replicate set of traps in each year. All catch data presented are the mean values ± standard error of the mean (SE) per trap over the length of each trial.

## 3. Results

Comparison of female and total moth catches in orchard blocks under various MD treatments was assessed in 2021 and 2022 (Table 1). Orchards treated with five MD tactics releasing sex pheromones, such as MEC sprayables, hand-applied dispensers, and aerosols were evaluated in six trials (trials #1–6) with 6 to 14 replicates (Table 2). In all trials, the female catch was significantly higher (2- to 6-fold) in traps baited with the CM4K, versus Combo + AA, lures. The total moth catch was significantly higher in traps with the CM4K versus Combo + AA lures (<3-fold) in five trials. Traps with the CM4K lure caught up to 11-fold more total moths than similar traps baited with the PH1X lure, and was significantly higher in all trials. Similarly, in four trials the total catch with the Combo + AA lure was significantly greater than with the PH1X lure.

**Figure 1:**
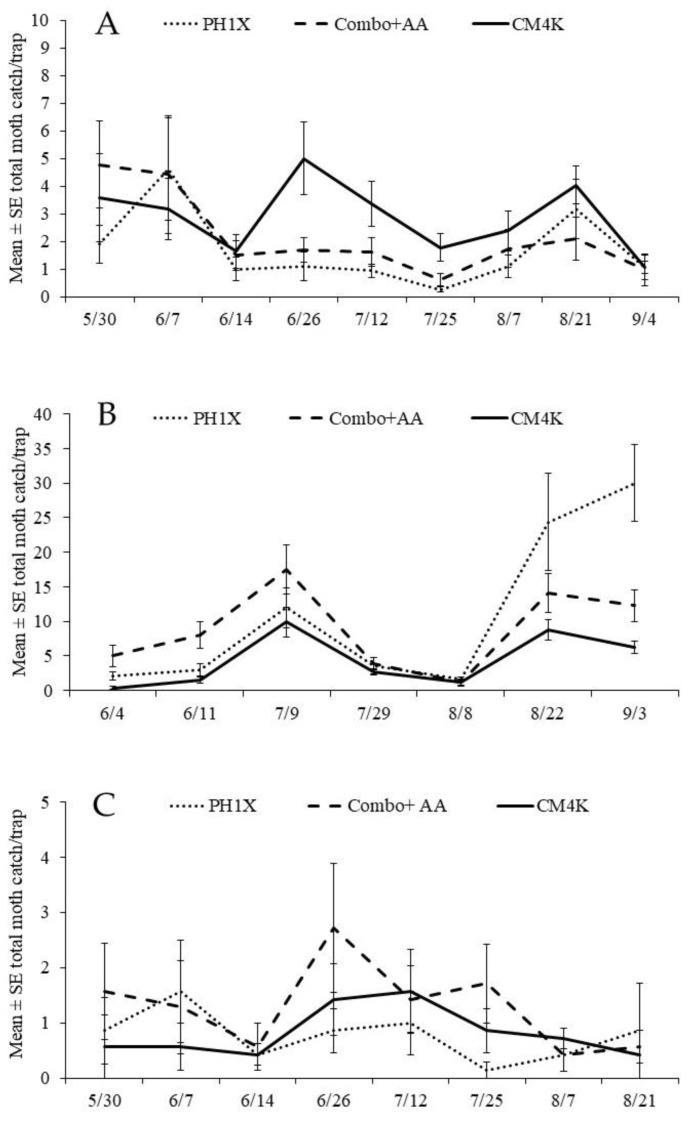
Seasonal catches of codling moth adults in traps with three different lures in (**A**) mowed apple, (**B**) weedy apples, and (**C**) pear crop, during 2021, in WA (USA).

The effectiveness of the CM4K lure versus the other two lures was also investigated in orchards treated with three dispensing systems releasing both sex pheromone and pear ester (trials #7–12) and in one trial (#13) with no mating disruption application (Table 3). In trial #13, in orchards without MD, there were no differences in the mean catch of females between CM4K and Combo + AA lures, and total catch was significantly lower with the PH1X than the other two lures. Traps with the CM4K lure caught significantly more females than the Combo + AA lure in all six trials under PH/PE MD, but total catch was significantly higher in only three trials (trials #7, #10, #13) (Figure 1A). Mean catch with the CM4K versus Combo + AA lures were ca. 2-fold higher with the CPD and MESO dispenser treatments and 9-fold higher with the PP dispensers, respectively. Total catch with the Combo + AA lure was significantly greater than with the PH1X lure in three trials (trials #9, #11,#13), while means with the CM4K lure were higher in all the trials ranging from 2 to 7-fold (Table 3).

**Figure 2:**
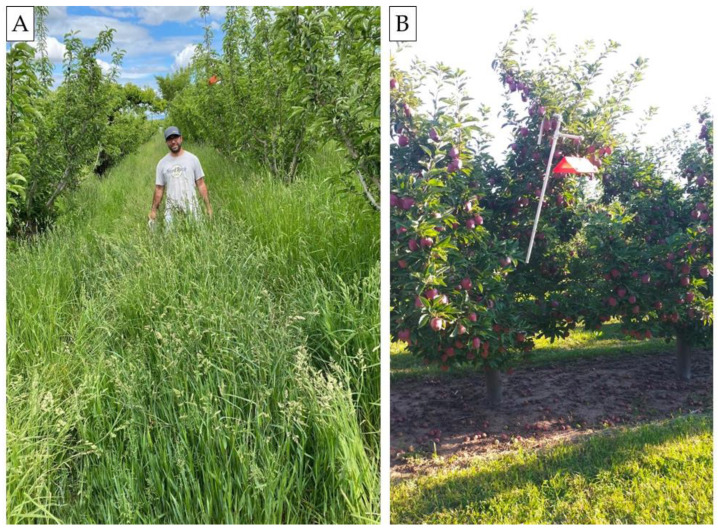
Photos of weed management in two organic apple orchards: (**A**) minimal weed management practices and (**B**) weed management (disking and mowing) in 2022, WA (USA).

In Tieton organic apple orchards treated with PP dispensers in 2021 (trial #14, Figure 1B), the relative performances of the three lures were found to be different than in other trials (Table 4). Despite similar significant differences being found for CM female catch between the CM4K and Combo + AA lures relative to previous trials, the total moth catches did not differ among the three lures (trial #14). Again in 2022, the same results were found in these orchards (trials #16, #17). The most obvious difference in the Tieton orchards compared with the other studies was their minimal use of weed management practices (Figure 2A). Specifically, very vigorous weed growth, with a height >30–50 cm, was found in these organic orchards, which were rarely mowed. In comparison, conventional apple orchards treated with herbicides and mowed between rows (weed height < 20 cm) (Figure 2B) had similar results among lures, as found in 2021 (Table 2 and Table 3). Weeds species were overall comparable across all study locations’ and comprised a complex of species belonging to Poaceae (*Lolium* spp., *Poa* spp., *Festuca* spp.), Plantaginaceae (*Plantago* spp.), Malvaceae (*Malva* spp.), Polygonaceae (*Rumex* spp.), Asteraceae (*Taraxacum* spp.) and Fabaceae (*Trifolium* spp.) families. The relative performance of the three lures in pear blocks treated with NoMate or PP dispensers in 2021 was similar to weedy apple orchards, but not to apple orchards which had been managed for weeds (Table 3, Figure 1C). Moth catches among lures in pear blocks with vigorous growth of the weeds were similar to weedy apple blocks, with similar weed growth (Table 4).

**Figure 3:**
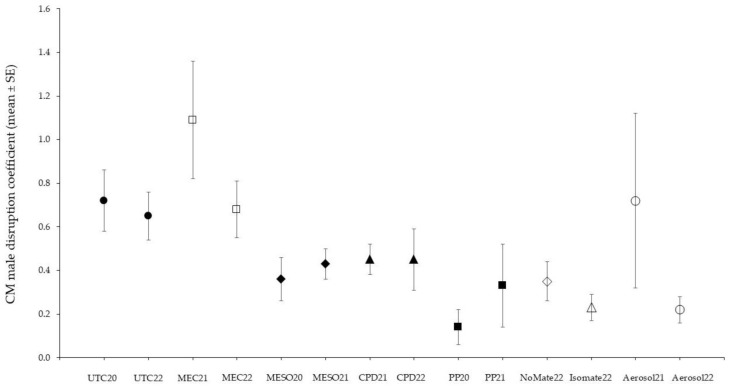
Comparison of codling moth male catch in traps baited with the PH1X versus the CM4K lures across different types of MD including untreated (UTC) -●-, sprayables MEC -□-, Meso -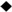-, CPD -▲-, PP -■-, NoMate -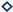
- and Isomate -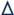
- dispensers, and aerosols -○- (Semios in 2021, Isomate in 2022).

The male disruption coefficient, defined as the ratio of males caught with the PH1X lure versus catches with the CM4K lure, varied about 10-fold under different MD programs (Figure 3). Interestingly, the ratio ranged somewhat higher in orchards treated with the MEC sprays than in untreated blocks. Values for the various MD programs using only sex pheromones versus sex pheromones in combination with pear ester were similar. The mean ± SE coefficient calculated from the weedy Tieton apple orchards was much higher (10-fold) than the blocks with standard weed management, with 2.72 ± 0.85 and 3.65 ± 0.65 in orchards treated with the CPD in 2021 and PP dispensers in 2022, respectively. Similarly, the coefficient was high, 3.48 ± 0.67 for the weedy pear orchards treated with the PP dispensers in 2022.

The same three lures were used to monitor five organic apple blocks treated with MESO and CPD dispensers and receiving weekly releases of sterile CM (trial #19) (Table 5). Female catch was significantly different and 4-fold higher with the CM4K than Combo + AA lures with both wild and sterile moths. Total moth catch did not differ for PH1X and Combo + AA lures for either wild or sterile moths. Total catch with the CM4K lure was significantly different, and 2-fold higher than the PH1X and Combo + AA, but not significantly different from the latter considering the sterile moths. The sterile/wild ratio was low across these orchards, at < 4.0. The highest S:W ratio was for total moths with the PH1X lure, and the lowest S:W ratio was with females caught with the CM4K lure.

**Figure 4:**
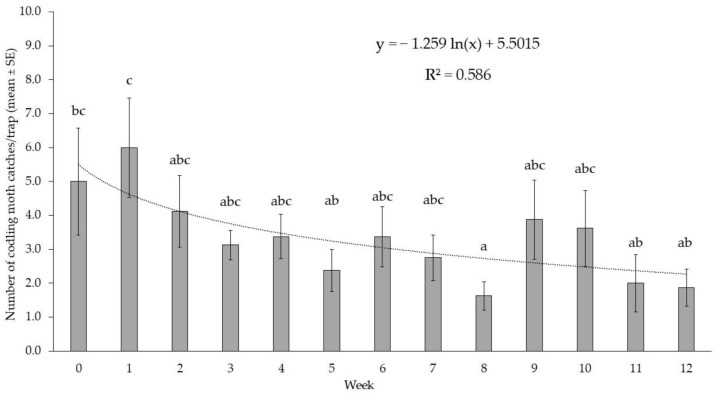
Total moth catches with CM4K lures of different ages (0- to 12-week-old) in delta traps (N = 8) within an apple orchard treated with CMDA PP dispensers in 2022 in WA (USA). Data were analyzed using a GLM function with Poisson distribution; columns with different letters are significantly different, with *p* < 0.05 by Tukey’s HSD test (df = 12, 91, *X*^2^ = 44.937, *p* < 0.001).

The performance of field-aged CM4K lures did not differ over 10 weeks (Figure 4). Lures aged for one week caught significantly more moths than lures aged 5, 8, 11 or 12 weeks. Traps baited with CM4K lures had a noticeable increase in mean CM catches at weeks 9 and 10, followed by a significant drop with older lures.

Two studies were conducted in 2022 to evaluate the impact of placing CM4K-baited traps near sex pheromone dispensers for MD. Male catch was significantly higher when traps baited with PH1X lure were placed 2.0–2.5 m from the PP dispensers than caught in traps with the CM4K lure placed adjacent to PP dispensers (trial #20, Table 6). There was no difference in female catch between the two distances using the CM4K lure. The total moth catch was significantly lower in traps baited with PH1X lure placed adjacent to PP dispensers compared with the same lure placed 2.0–2.5 m from them. The total moth catch with the CM4K lure was significantly higher than that of PH1X lure placed adjacent to PP, and did not differed between the two distances and from the PH1X lure placed 2.0–2.5 m from the PP dispensers.

Traps baited with either the CM4K or PH1X lures spaced 10 m from Isomate CM Mist aerosol dispensers had significantly different male and total catches (trial #21, Table 6). In comparison, moth catches in traps with each lure, but spaced at 35 m from the aerosol, were not significantly different in male and total catches. Female catches with the CM4K lure were not affected by the trap’s distance from the aerosol unit.

**Figure 5:**
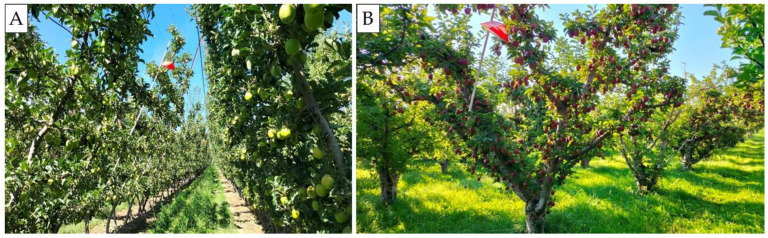
Photos of (**A**) a young, trellised spindle orchard with typical weed management in the tree row and of (**B**) a mature apple orchard with minimal weed management used to evaluate trap placement near fruit clusters in 2022, WA (USA).

Two trials evaluating the performance of the CM4K relative to the proximity to apple fruits in 2022 had different results (Table 7, Figure 5). In a young, trellised spindle apple orchard, traps placed near groups of fruit (5–9 fruits) had significantly greater catch rates for CM females and lower male catch than traps not placed near fruit (trial #22, Figure 5A). Placing traps near a smaller cluster of fruit had an intermediate impact on moth catch. The second experiment was conducted in a closed canopy of large apple trees with traps placed either ≤2 m from clusters of at least 10 fruits or at a distance of >2 m from the nearest fruit cluster (trial #23, Figure 5B). No difference was found in moth catch in the CM4K-baited traps at these two distances and fruit load (Table 7).

## 4. Discussion and Conclusions

The CM4K lure was very effective under a range of MD programs tested over the two-year study. In some cases, the total moth catch was similar between the Combo + AA or the CM4K lures, but generally the latter caught significantly greater numbers of female moths. In orchards untreated with MD, the two lures performed similarly. There was no apparent difference in lure effectiveness under either PH or PH/PE MD programs.

The CM4K lure was significantly more effective than sex pheromone lures when placed near hand-applied dispensers or aerosol emitters. A previous study conducted in one area-wide management site in north-central Washington with 318 traps reported that the mean distance of sex pheromone-baited traps was only 0.8 m from Isomate C+ dispensers [26]. Adoption of the CM4K lure could diminish the potential disruption of the trap by sex pheromone dispensers either at a high (1000 units ha^−1^) or low (1 unit ha^−1^) dispenser density.

The CM4K lure has several characteristics which can aid CM management. This non-pheromone lure performed better or equivalently to the widely used sex pheromone–pear ester-based lure. Unlike sex pheromone lures, the CM4K is not impacted by the trap’s proximity to MD dispensers. Trap placement should always consider the influence of canopy position and proximity to fruits which might interact with moth orientation into traps. For instance, in a young, trellised spindle apple orchard, more CM females were caught in traps close (≤1 m) to groups of fruits compared to areas with no fruits within the same distance range, while significantly more CM males were caught in the absence of fruits. Nevertheless, this result was not confirmed in an old central leader apple orchard with high fruit load.

The CM4K lure was effective with both wild and sterile CM. Lures with sex pheromone may incur overestimations of the S:W ratio due their greater drawing radius [23]. While using lures that catch more sterile moths could influence the effectiveness of the program and likely require more frequent liner replacements, the most important aspect of using the CM4K lure could be its ability to detect wild females and foretell local fruit damage based on female moth density [23].

The longevity of the CM4K lure was not significantly different over the 10 weeks the lures were aged, plus the additional 3-week assessment period. However, there was an initial bump in moth catch with new lures and lures only 1-week old, followed by a gradual decline over time. Previously, the AA lure was found to last the entire season with a very consistent release [11]. DMNT was reported to be much more short-lived than pear ester from a gray halobutyl septum [45]. The emission rates of linalool oxide from either a PVC or septa-based lure have not been reported. Unless repellent secondary compounds are formed as the principal volatiles age inside the lure, the four-component CM4K lure will eventually degrade to a three- or two-component lure, which are still both highly attractive [13].

Developing the use of a host plant volatile for the monitoring and management of an important pest requires a variety of studies to elucidate the factors impacting its effectiveness [28]. Pear ester, discovered to be a potent adult and larval attractant for CM in 1999, has since been extensively studied, and a variety of management tools have been developed and adopted by growers for CM management [46]. Similarly, the development of the CM4K lure has required that its performance to be broadly characterized. Variable results in Italy and eastern North America compared with our studies in the western USA were consistent with the variability found with pear ester among regions [46]. However, some new factors not previously considered with pear ester became apparent in assessing the effectiveness of the CM4K lure. A description of weed management was not reported in any of the initial studies evaluating pear ester. Weed management is not something that entomological research on orchard tortricids has previously addressed.

Subtle factors can influence the performance of individual traps and contribute to trap-to-trap variability [24]. Monitoring CM with pear ester was effective across major apple cultivars but seemed to work best in walnut, and then apple, and was least attractive in pear [3,9,27]. Higher-load pear ester lures caught more CM in pear orchards [9]. Studies of apple also demonstrated that placing traps baited with pear ester adjacent to fruits reduced moth catch [28]. In contrast, with the CM4K lure, catch rates of female CM were enhanced when traps were placed near clusters of fruit in an open trellised spindle canopy. Interestingly, male catch was lower in these traps. Monitoring CM in a mature closed canopy central leader orchard, likely with more uniform host volatiles at a higher concentration did not suggest trap placement with the CM4K lure was important.

Weed management in orchards can involve both chemical herbicides and cultural practices [47]. Weed competition, especially with young trees, is a key agronomic concern [38,48]. One focus has been to manage weedy plant species in orchards to aid the conservation of biological control agents at the population level without impacting the tree’s physiology [49,50]. Identification of general volatiles attractive to natural enemies and the development of attractive lures to bolster biological control in orchards has also been researched [51]. The contribution of volatile emissions from weeds in creating a background profile within orchards, and their impact on pests and natural enemies has rarely been reported [52]. One study detailed a host shift due to the interplay of volatiles emitted by ground-cover aromatic weeds and an apple host with the lyonetiid leafminer, *Lyonetia prunifoliella* (Hübner) [53]. No similar studies have been reported for tortricids. Some tortricids can be found utilizing ground-cover plants within a perennial host planting [54,55]. Chemical ecological studies with this important taxonomic group have focused mostly on either the impact of host volatiles on the effectiveness of sex pheromone lures [56], or the release of herbivore-induced volatiles and the development of lures for mostly leaf-feeding species [46,57].

The discovery of the CM4K lure was made in minimally sprayed, conventionally managed ‘Delicious’ apple blocks, but with a standard chemical weed removal program, which were mowed regularly during the season [2]. Lures performed similarly in blocks with heavy or light fruit loads, with high levels of injured fruit, and were still effective late in the season when the ground was littered with fermenting CM-injured fruits. In contrast, weed management within orchards where the CM4K lure was tested in Italy were not reported [16,30]. Many of the blocks were organic, and were chosen because they had ‘good’ populations of CM. It is likely that the difficulty in managing CM and weeds in organic orchards are correlated.

The background volatile profile in weedy orchards is more likely a blend from host and non-host plants. Several ubiquitous host volatiles were found to reduce male and female CM catches in traps, such as (*Z*)-3-hexenol, (*E*)-2-hexanal, (*Z*)-3-hexenyl acetate, linalool, methyl salicylate, and hexyl butanoate when added to the pear ester + DMNT + AA lure [18]. These same six compounds are key odorants released by grasses and broadleaf weeds, especially when damaged by mowing or herbivory [58,59]. They are also key odorants of apple [60,61]. A second factor impacting the lure’s performance in weedy orchards could come from elevated levels of other ubiquitous volatiles that are attractants for CM, such as DMNT, (*E*,*E*)-α-Farnesene, or β-Myrcene. Traps baited with a sex pheromone or an attractive allelochemical lure placed in a host crop but with a distorted background blend of volatiles can be masked [35,52]. This effect appeared to occur more strongly for male than female CM in the weedy orchards.

The Tieton orchards had minimal weed management and relied on workers walking with gas-powered “weed eaters” to free the under-tree sprinklers from plant growth twice per year. The drive rows in the orchards were also mowed only twice per year. The specific impact of the timing of these weed management practices on the CM4K lure was not addressed in our studies. The range of weed densities and species observed in orchards outside of the Tieton area were typical of central Washington orchards incorporating a mix of herbicide and cultural practices [62]. The relative effectiveness of the CM4K lure in orchards with a range of intermediate levels of seasonal weed management is unknown. Yet, our data suggest that competition with non-host volatiles in orchards should be considered in any future development of allelochemical lures used for monitoring or mass-trapping female moths.

Traps baited with sex pheromone lures for CM catch very low numbers of *Grapholita molesta* (Busck), <1% than what caught with their own sex pheromone [45]. One report suggested that the CM4K lure may be attractive to *G. molesta* in apple orchards not treated with MD in Massachusetts, though they did not compare it with any OFM lure and the proportion of males in the catch over three time periods during the season was 0.97 [31]. Several studies conducted with *G. molesta* in Washington apple orchards found that the catch with the CM4K lure was <2% of concurrent catches with the PHEROCON^®^ OFM Combo Dual lure (Trécé Inc.) [63]. Interestingly, the proportion of males caught with the CM4K lure in Washington was only 0.53. However, an intermediate result was reported from a single orchard in Uruguay treated with aerosols, where the CM4K lure did catch about 20% as many *G. molesta* as the PHEROCON^®^ OFM Combo Dual lure, and the sex ratio of males was 0.80. There is no information about possible attraction of *G. molesta* adults to the three plant volatiles in the lure. However, we found that the same AA lure was not attractive to *G. molesta* [45]. The possible cross contamination of CM traps with *G. molesta* sex pheromone cannot be discounted in field studies with both species.

The male disruption coefficient is intended to be a useful indicator of male disruption under different MD programs. The coefficient varied among MD treatments, suggesting they may differ in their effectiveness. However, the coefficient was not a useful measure in weedy orchards where the allelochemical lure was strongly impacted. The low catch of males in traps with the PH1X lure is impacted both by CM adult density and the level of communication disruption [64]. Interestingly, the CM MEC treatments had the highest coefficients, suggesting they might not be as effective and/or they might differentially attract CM males into the treated blocks compared with other dispensing systems.

The coefficients calculated with the different dispensing systems may suggest different primary mechanisms of CM disruption and their level of effectiveness. Low-volume spray applications of MEC formulations create thousands of supercharged leaves that can serve as “female-equivalent” sources for male CM within the orchard [65]. In contrast, the application of mating disruption dispensers (Isomate C+) for CM has been shown to pull moths at least to the edge of the treated orchard [25]. Close orientation to hand-applied dispensers within the orchard can incapacitate males from finding females or entering traps [66]. Among the five hand-applied dispensers the three with the highest densities (800–1000 ha^−1^) had lower coefficients compared with the MESO (80 ha^−1^) and CPD (10 ha^−1^) dispensers. Aerosol devices thought to achieve disruption similarly [67] had variable and intermediate coefficients.

A clearer interpretation of these male-based coefficients under different MD dispensing systems would be strengthened with data on the relative proportions of mated female CMs. Previously, we found that proportions of mated female CMs caught with the CM4K lure were high in orchards under several MD programs [17]. These data were consistent with the use of light and interception traps and pear ester lures in MD-treated orchards [68,69,70]. Female mating levels would be useful in evaluating various programs, such as the ones presented here, and to assess more intensive MD tactics that either increase dispenser densities or combine different tactics. The addition of a solar-powered UV-A LED light to delta traps has significantly increased the female catch rates of several tortricids, including CMs, and could be used to more readily assess female mating under different variants of MD tactics [19,63]. Studies are needed to assess whether CM4K provides an accurate estimate of female mating in a population, or if it is biased for either mated or unmated females.

## Figures and Tables

**Table 1:** Summary of lure studies conducted in apple and pear orchards during 2020–2022.

Table or Figure	Trial #	Year	MD Treatment a	Replicates #	Effect on Lure Performance
Table 2	1–6	2021–2022	MEC, Isomate, NoMate, aerosols	6–14	PH-based MD
Table 3	7–13	2021–2022	PP, CPD, MESO, none	6–14	PH/PE-based MD, no MD
Table 4	14–18	2021–2022	PP, NoMate	8–11	Weed management, apple and pear
Table 5	19	2021	PP	10	Wild and sterile moths
Table 6	20–21	2022	PP	10	Trap’s distance from MD dispensers
Table 7	22–23	2022	PP, aerosol	10, 21	Trap’s proximity to fruit
Figure 1	-	2021	PP, NoMate	8–10	Seasonal catches in apple, pears, and weedy plots
Figure 2	-	2022	-	-	Photos of orchards with weeds and no weeds
Figure 3	-	2020–2022	none, various ^a^	4–10	Coefficient of CM male disruption
Figure 4	-	2022	PP	8	Longevity of CM4K lure
Figure 5	-	2022	-	-	Photos of trellised and central leader canopies

^a^ MD treatments included two aerosol cannisters, Semios CM Aerosol and Isomate CM Mist, applied at 2.5 units ha^−1^; three sex pheromone dispensers, NoMate CM, Isomate CM Flex, and CIDETRAK CM PP, applied at 1000 dispensers ha^−1^; and a microencapsulated sprayable, CIDETRAK CM MEC, releasing only sex pheromone (PH); two commercial dispensers loaded with codlemone and pear ester, CIDETRAK CMDA COMBO MESO-A, applied at 80 ha^−1^, and CIDETRAK CMDA COMBO PP, applied at 500–1000 ha^−1^; and the experimental CPD dispenser, applied at 10 units ha^−1^ (PH/PE).

**Table 2:** Comparison of three lures (PH1X, Combo + AA, and CM4K) for female and total codling moth mean catch (± SE) in delta traps in replicated orchards (N = 6–14) under several sex pheromone-based MD programs during 2021–2022 in WA, USA.

Lures	Moths	Trial #1, CMMEC 2021 (N = 12)	Trial #2, Semios Aerosol 2021 (N = 6)	Trial #3, CM MEC 2022 (N = 14)	Trial #4, Isomate CM 2022 (N = 12)	Trial #5, NoMate CM 2022 (N = 9)	Trial #6, Isomate Aerosol 2022 (N = 9)
Combo + AA	Females	1.0 ± 0.2 b	4.3 ± 1.5 b	1.5 ± 0.3 b	19.7 ± 4.5 b	38.4 ± 11.0 b	4.2 ± 1.1 b
CM4K	Females	6.1 ± 1.0 a	16.3 ± 3.7 a	7.5 ± 1.9 a	41.1 ± 9.9 a	71.7 ± 23.6 a	10.6 ± 3.2 a
	Statistics	*F*_1,22_ = 36.29 *p* < 0.0001	*F*_1,10_ = 11.12 *p* = 0.0076	*X^2^* = 62.24 *p* < 0.001	*X^2^* = 92.58 *p* < 0.001	*X^2^* = 91.63 *p* < 0.001	*X^2^* = 25.24 *p* < 0.001
PH1X	Total	5.0 ± 1.1 b	11.0 ± 4.8 b	11.6 ± 4.0 b	13.4 ± 2.9 c	12.0 ± 1.8 c	2.1 ± 0.5 b
Combo + AA	Total	4.7 ± 1.2 b	15.2 ± 3.0 b	6.6 ± 1.5 c	65.4 ± 14.3 b	112.9 ± 28.8 b	17.8 ± 3.2 a
CM4K	Total	10.5 ± 1.5 a	40.8 ± 8.2 a	19.4 ± 5.0 a	89.8 ± 17.4 a	136.0 ± 42.9 a	25.8 ± 6.2 a
	Statistics	*F*_2,33_ = 6.16 *p* = 0.0053	*F*_2,15_ = 7.84 *p* = 0.0047	*X^2^* = 93.71 *p* < 0.001	*X^2^* = 92.58 *p* < 0.001	*X^2^* = 1197.7 *p* < 0.001	*F*_2,24_ = 18.53 *p* = 0.0047

Normalized data were subjected to ANOVA (F-statistic) using the LM function, while data which could not be normalized were analyzed using a GLM function with Poisson distribution; means followed by different letters within column (separated for females and total) are significantly different, *p* < 0.05, Tukey’s HSD test.

**Table 3:** Comparison of three lures (PH1X, Combo + AA, and CM4K) for female and total codling moth mean catch (±SE) in delta traps in replicated orchards (N = 6–12) under several MD programs utilizing both sex pheromone and pear ester and without MD (no MD) during 2021–2022 in WA, USA.

Lures	Moths	Trial #7, CPD 2021 (N = 6)	Trial #8, CPD 2022 (N = 8)	Trial #9, MESO 2020 (N = 6)	Trial #10, MESO 2021 (N = 10)	Trial #11, MESO 2022 (N = 14)	Trial #12, PP 2021 (N = 12)	Trial #13, No MD 2022 (N = 9)
Combo + AA	Females	4.3 ± 1.5 b	0.9 ± 0.4 b	8.0 ± 2.7 b	3.6 ± 0.5 b	3.1 ± 1.4 b	0.5 ± 0.2 b	14.4 ± 5.1 a
CM4K	Females	16.3 ± 3.7 a	3.4 ± 0.8 a	29.0 ± 5.8 a	15.7 ± 3.0 a	10.0 ± 3.6 a	5.0 ± 1.2 a	34.0 ± 15.2 a
	Statistics	*F*_1,10_ = 11.12 *p* = 0.0076	*F*_1,14_ = 11.93 *p* = 0.0039	*F*_1,10_ = 14.93 *p* = 0.0031	*F*_1,18_ = 25.21 *p* = 0.0001	*X^2^* = 54.14 *p* < 0.001	*X^2^* = 51.28 *p* < 0.001	*F*_1,16_ = 0.98 *p* = 0.3358
PH1X	Total	11.0 ± 4.8 b	0.9 ± 0.5 b	10.5 ± 3.7 b	8.0 ± 1.2 b	3.5 ± 0.8 c	1.4 ± 0.8 b	34.4 ± 13.0 b
Combo + AA	Total	15.2 ± 3.0 b	2.6 ± 1.3 ab	34.3 ± 5.6 a	14.6 ± 2.1 b	10.3 ± 3.7 b	1.1 ± 0.4 b	77.4 ± 33.5 a
CM4K	Total	40.8 ± 8.2 a	6.3 ± 1.9 a	59.0 ± 10.9 a	36.4 ± 5.1 a	21.6 ± 7.8 a	9.0 ± 2.3 a	82.7 ± 34.3 a
	Statistics	*F*_2,21_ = 7.84 *p* = 0.0047	*F*_2,21_ = 6.25 *p* = 0.0074	*F*_2,15_ = 15.62 *p* = 0.0002	*F*_2,27_ = 25.93 *p* < 0.0001	*X^2^* = 205.64 *p* < 0.001	*X^2^* = 117.65 *p* < 0.001	*X^2^* = 217.13 *p* < 0.001

See footnote of Table 2.

**Table 4:** Comparison of three lures (PH1X, Combo + AA, and CM4K) for female and total codling moth catch in delta traps in replicated apple and pear orchards (N = 8–11) without weed management in blocks or with the use conventional or organic practices in apple and pear during 2021–2022 (18 May–13 September 2021 and 23 May–4 September 2022) in WA, USA.

Lures	Moths	Mean ± SE Catches per Trap				
		Trial #14 ^a^, Weeds Apple 2021 (N = 8)	Trial #15 ^b^, No Weeds Apple 2021 (N = 8)	Trial #16 ^a^, Weeds Apple 2022 (N = 11)	Trial #17 ^a^, Weeds Pear 2022 (N = 10)	Trial #18 ^b^, No Weeds Pear 2022 (N = 9)
Combo + AA	Females	7.5 ± 1.9 b	4.0 ± 0.5 b	7.0 ± 0.8 b	8.9 ± 1.8 b	4.2 ± 1.2 a
CM4K	Females	23.1 ± 6.2 a	25.3 ± 2.3 a	18.4 ± 2.7 a	28.1 ± 5.1 a	6.8 ± 2.8 a
	Statistics	*F*_1,14_ = 8.20 *p* = 0.0133	*F*_1,14_ = 120.86 *p* < 0.0001	*F*_1,42_ = 14.99 *p* = 0.0004	*F*_1,18_ = 13.72 *p* = 0.0016	*F*_1,16_ = 0.29 *p* = 0.5996
PH1X	Total	59.1 ± 15.9 a	21.9 ± 3.9 b	55.7 ± 9.8 a	60.8 ± 12.7 a	6.2 ± 2.1 a
Combo + AA	Total	72.4 ± 13.2 a	35.8 ± 7.6 b	49.5 ± 5.8 a	63.2 ± 7.1 a	16.6 ± 5.8 a
CM4K	Total	56.3 ± 21.3 a	73.3 ± 10.2 a	36.7 ± 4.9 a	48.7 ± 7.9 a	14.0 ± 5.9 a
	Statistics	*F*_2,21_ = 0.38 *p* = 0.6891	*F*_2,21_ = 13.98 *p* = 0.0001	*F*_2,63_ = 1.50 *p* = 0.2304	*F*_2,27_ = 0.76 *p* = 0.4794	*F*_2,24_ = 1.35 *p* = 0.2786

Normalized data were subjected to ANOVA (F-statistic) using LM function and means followed by different letters within columns (separated for females and total) and are significantly different at *p* < 0.05, Tukey’s HSD test. ^a^ MD treatment in weedy apple and pear orchards near Tieton (WA) used CIDETRAK CMDA PP dispensers at 800 ha^−1^. ^b^ Apple and pear replicates with weed management used organic blocks near Wapato (WA) treated with NoMate CM dispensers at 1000 ha^−1^.

**Table 5:** Monitoring wild and released sterilized codling moth in organic apple orchards (N = 10) receiving weekly releases of 2000 sterilized moths ha^−1^ during 2021 (6 May–13 August 2021) in WA (USA).

Trial #19	Mean ± SE Moth Catch per Trap per Season				Sterile/Wild Overflooding Ratio	
	Females		Total			
Lure	Sterile	Wild	Sterile	Wild	Females	Total
PH1X	-	-	73.4 ± 24.1 b	18.2 ± 8.9 b	-	4.03
Combo + AA	17.5 ± 2.3 b	7.5 ± 1.3 b	79.9 ± 15.8 b	20.5 ± 4.0 b	2.33	3.90
CM4K	64.5 ± 9.6 a	31.0 ± 5.8 a	154.4 ± 28.6 a	54.2 ± 10.1 a	2.08	2.85
Statistics	*F*_1,18_ = 28.75 *p* < 0.0001	*F*_1,18_ = 26.40 *p* = 0.0001	*F*_2,27_ = 4.03 *p* = 0.0293	*F*_2,27_ = 8.43 *p* = 0.0014	-	-

Normalized data were subjected to ANOVA (F-statistic) using an LM function, and the means followed by different letters within columns are significantly different, with *p* < 0.05 by Tukey’s HSD test.

**Table 6:** Proximity of lure-baited traps to CM MD dispensers in conventional apple orchards treated with CIDETRAK CMDA PP dispensers (1000 ha^−1^) and Isomate CM Mist aerosol (2.5 ha^−1^).

Trial	Dispensers	Lure	Distance (m) from Dispenser	Mean ± SE Moths per Trap		
				Males	Females	Total
#20	CMDA PP	PH1X	≤0.5	0.7 ± 0.2 b	-	0.7 ± 0.2 b
			2.0–2.5	2.5 ± 0.7 a	-	2.5 ± 0.7 a
		CM4K	≤0.5	0.8 ± 0.4 b	2.0 ± 0.4 a	2.8 ± 0.6 a
			2.0–2.5	1.2 ± 0.3 ab	2.3 ± 0.4 a	3.5 ± 0.5 a
			Statistics	*X^2^* = 14.31 *p* = 0.0025	*X^2^* = 0.21 *p* = 0.647	*X^2^* = 21.82 *p* < 0.001
#21	CM Mist	PH1X	10	1.9 ± 0.7 b	-	1.9 ± 0.7 b
			35	9.7 ± 2.3 a	-	9.7 ± 2.3 a
		CM4K	10	14.6 ± 3.6 a	7.3 ± 1.2 a	21.9 ± 4.2 a
			35	10.0 ± 1.8 a	9.7 ± 1.6 a	19.7 ± 3.1 a
			Statistics	*F*_3,24_ = 7.76 *p* = 0.0009	*F*_1,12_ = 1.20 *p* = 0.2950	*F*_3,24_ = 16.12 *p* < 0.0001

Normalized data were subjected to ANOVA (F-statistic) using an LM function, while data which could not be normalized were analyzed using a GLM function with Poisson distribution; means followed by different letters are significantly different, with *p* < 0.05 by Tukey’s HSD test.

**Table 7:** Moth catches in delta traps baited with a CM4K lure categorized by the number of apple fruits within 1.0 m of the trap in a 5-year-old trellised slender spindle conventional apple orchard with trees planted at spacings of 0.5 × 3.7 m and treated with CIDETRAK CM PP dispensers (trial #22, N = 21), or in a 30-year-old organic apple orchard with a closed canopy with a heavy fruit set that had not been thinned, and where apple fruits were planted as a central leader at 4.3 × 4.3 m and treated with CIDETRAK CM PP dispensers (trial #23, N = 10).

Trial	# Fruits Within Distance from Trap	Mean ± SE Moths per Trap		
		Males	Females	Total
#22	None ≤ 1 m	33.3 ± 4.4 a	6.0 ± 0.8 b	39.3 ± 4.4 a
	1–4 ≤ 1 m	28.8 ± 3.2 a	9.7 ± 1.5 ab	38.6 ± 3.6 a
	5–9 ≤ 1 m	20.2 ± 2.7 b	12.8 ± 1.9 a	33.0 ± 3.4 a
	Statistics	*F*_2,60_ = 4.34 *p* = 0.0173	*F*_2,60_ = 5.92 *p* = 0.0045	*F*_2,60_ = 0.82 *p* = 0.4465
#23	None ≤ 2 m	10.5 ± 3.0 a	7.1 ± 1.3 a	17.6 ± 4.1 a
	>10 ≤ 2 m	9.9 ± 1.7 a	8.9 ± 1.7 a	18.8 ± 3.2 a
	Statistics	*F*_1,18_ = 0.05 *p* = 0.8184	*F*_1,18_ = 0.83 *p* = 0.3751	*F*_1,18_ = 0.18 *p* = 0.6750

Normalized data were subjected to ANOVA (F-statistic) using LM function, and means followed by different letters are significantly different, with *p* < 0.05 by Tukey’s HSD test.

## Data Availability

The original contributions presented in this study are included in the article. Further inquiries can be directed to the corresponding author.
